# Sulforaphane Reverses the Expression of Various Tumor Suppressor Genes by Targeting DNMT3B and HDAC1 in Human Cervical Cancer Cells

**DOI:** 10.1155/2015/412149

**Published:** 2015-06-16

**Authors:** Munawwar Ali Khan, Madhumitha Kedhari Sundaram, Amina Hamza, Uzma Quraishi, Dian Gunasekera, Laveena Ramesh, Payal Goala, Usama Al Alami, Mohammad Zeeshan Ansari, Tahir A. Rizvi, Chhavi Sharma, Arif Hussain

**Affiliations:** ^1^Department of Natural Science and Public Health, College of Sustainability Sciences & Humanities, Zayed University, P.O. Box 19282, Dubai, UAE; ^2^School of Life Sciences, Manipal University, P.O. Box 345050, Dubai, UAE; ^3^Department of Microbiology & Immunology, Faculty of Medicine and Health Sciences, United Arab Emirates University, P.O. Box 17666, Al Ain, UAE

## Abstract

Sulforaphane (SFN) may hinder carcinogenesis by altering epigenetic events in the cells; however, its molecular mechanisms are unclear. The present study investigates the role of SFN in modifying epigenetic events in human cervical cancer cells, HeLa. HeLa cells were treated with SFN (2.5 *µ*M) for a period of 0, 24, 48, and 72 hours for all experiments. After treatment, expressions of DNMT3B, HDAC1, RAR*β*, CDH1, DAPK1, and GSTP1 were studied using RT-PCR while promoter DNA methylation of tumor suppressor genes (TSGs) was studied using MS-PCR. Inhibition assays of DNA methyl transferases (DNMTs) and histone deacetylases (HDACs) were performed at varying time points. Molecular modeling and docking studies were performed to explore the possible interaction of SFN with HDAC1 and DNMT3B. Time-dependent exposure to SFN decreases the expression of DNMT3B and HDAC1 and significantly reduces the enzymatic activity of DNMTs and HDACs. Molecular modeling data suggests that SFN may interact directly with DNMT3B and HDAC1 which may explain the inhibitory action of SFN. Interestingly, time-dependent reactivation of the studied TSGs via reversal of methylation in SFN treated cells correlates well with its impact on the epigenetic alterations accumulated during cancer development. Thus, SFN may have significant implications for epigenetic based therapy.

## 1. Introduction

Genetic alterations, such as mutations and aberrant epigenetic regulation, lead to susceptibility to develop cancer [1, 2]. Notably, reversible nature of epigenetic machinery makes it an attractive molecular target for cancer prevention and can be achieved by understanding the initiation and maintenance of epigenetic gene silencing [3, 4]. Extensive studies have shown that promoter hypermethylation is one of the imperative mechanisms for epigenetic-mediated inactivation of numerous tumor suppressor genes (TSGs) during the development of cancer [4–6]. DNA methyltransferases (DNMTs) facilitate hypermethylation of CpG islands in the promoter region of TSGs leading to silencing of these genes either by checking the binding of transcription factors or by facilitating methylated DNA-binding proteins which further recruit other transcriptional repressors such as histone deacetylases (HDACs) and histone methyl transferases, subsequently resulting in transcriptionally inactive chromatin form [5, 7].

Synthetic epigenetic drugs (e.g., Aza-deoxycytidine, trichostatin A) that regulate epigenetic patterns including DNA methylation and histone modification, via key targeting enzymes, namely, DNA methyltransferases and histone deacetylases, are under clinical studies [8–10]. However, certain disadvantages such as lack of specificity, short duration of action, and unpredicted effects on functional and structural patterns of normal cells restrict the general use of these synthetic epigenetic drugs [8–10]. Nowadays, research on plant derived dietary factors is gaining more attention to utilize them as epigenetic modifiers since these agents have been found to revert the aberrant epigenetic patterns with the least undesirable traits which are caused by available synthetic epigenetic drugs.

Use of dietary phytochemicals including SFN, genistein, curcumin, resveratrol, and EGCG provides a promising prospect for the development of better and effective strategy to prevent the cancer risk as these agents can block or reverse cancer progression through various molecular targets including epigenetic modulation [11–15]. SFN, an isothiocyanate, is the main bioactive constituent of cruciferous vegetables such as broccoli, cabbage, garden cress, cauliflower, and Brussels sprouts. Many studies have shown its antibacterial, antiproliferative, anti-inflammatory, antiangiogenic, and antimetastatic effects, differentiation- or apoptosis-inducing effect, and ability to arrest the cell cycle, inhibit DNA adduct formation, and modulate phase I and II xenobiotic-metabolizing enzymes and antioxidant activities [16–18]. Given this context of chemopreventive potential of SFN as an epigenetic modifier, the present study was designed to investigate inhibition of DNMTs and HDACs by SFN and its effect on the expression of epigenetically modified (hyper- or hypomethylated) genes including RAR*β*, CDH1, DAPK1, and GSTP1 in human cervical cancer cell line, HeLa. To correlate the inhibition of DNMTs and HDACs activity induced by SFN, we performed* in silico* molecular modelling and docking studies on DNMT3B and HDAC1.

## 2. Materials and Methods 

### 2.1. Cell Line and Cell Culture

The human cervical carcinoma cell line, HeLa, was maintained in DMEM (Sigma, USA) supplemented with 10% heat inactivated fetal bovine serum and 100 *μ*g/mL penicillin-streptomycin (Sigma, USA) and maintained in a humidified atmosphere of 5% CO_2_ in air at 37°C.

### 2.2. Chemicals and Reagents

SFN, trichostatin A (TSA), and 5-Aza-deoxycytidine (5-Aza-dC) were obtained from Sigma (Sigma, USA). Nuclear extraction kit, DNMTs, and HDACs activity assay kit were purchased from Epigentek (Epigentek, USA). DNA purification (GenElute Mammalian Genomic DNA), total RNA purification (GenElute Mammalian Genomic Total RNA), and DNA modification (Imprint DNA Modification) kits were purchased from Sigma (Sigma, USA). RT-PCR (ProtoScriptM-MuLVTaq) kit was obtained from New England Bio Labs (New England Bio Labs, USA).

### 2.3. Preparation of Drugs

A stock solution of SFN (10 mM) was prepared in DMSO (Sigma, USA). The solution was stored in aliquots at −20°C. Further dilution was made in a complete medium (DMEM supplemented with 10% FBS) to the required concentrations of 2.5 *μ*M for the treatment of HeLa cells. A substock of 500 *μ*M TSA was prepared from 5 mM stock solution. A working concentration of 0.05 *μ*M was further used for the experiments. A stock solution of 219 mM 5-Aza-deoxycytidine (5-Aza-dC) (Sigma, USA) was prepared and further working concentration of 1.5 *μ*M was made from 10 mM substock.

### 2.4. DNMT Activity Assay

HeLa cells were treated with SFN and 5-Aza-dC for 3 days. The cells were then harvested and nuclear extracts were prepared from treated cells according to various time points using EpiQuik nuclear extraction kit (Epigentek, USA) as per the manufacturer's protocol. DNMT activity was then assayed using the EpiQuik DNMT activity assay kit (Epigentek, USA) as per protocol instructions.

### 2.5. HDAC Activity Assay

The effect of SFN on HDAC activity in HeLa cells was determined using the EpiQuik HDAC activity assay kit (Epigentek, USA) according to the manufacturer's instructions. Briefly, HeLa cells were treated with SFN and TSA for 3 days and harvested and nuclear extracts were then prepared for the same using EpiQuik nuclear extraction kit (Epigentek, USA) following the manufacturer's instructions.

### 2.6. *In Silico* Molecular Modeling Studies of DNMT3B and HDAC1

To address the interaction of SFN with the epigenetic modulator enzymes DNMT3B and HDAC1, the 3D structures of the proteins were required. Complete structure of DNMT3B or its catalytic domain is currently not available in structure databases [19]. Though a part of the DNMT3B structure is solved, it comprises only the PWWP domain that lies in the N-terminal region of the protein away from the C-terminal catalytic domain. Hence, there was a need to produce a homology model of the catalytic domain of DNMT3B. The protein sequence of DNMT3B (UniProt ID: Q9UBC3) was retrieved from UniProt Knowledgebase (UniProtKB) [20]. The BLAST tool on NCBI was used to identify the possible structural template for the catalytic domain of DNMT3B [21]. The BLAST search was performed with the full DNMT3B sequence as query and PDB as the target database with default parameter. The top hit was the catalytic domain of DNMT3A (RCSB Protein Data Bank; PDB ID: 2QRV) which gave the best coverage (33%) for the full DNMT3B sequence with an *e*-value of 5*e* − 169 and 80% sequence identity. The template covered from residue 568 to 852 of DNMT3B which includes the full catalytic domain [22]. The SWISS-MODEL homology modelling server was used to model the catalytic domain of DNMT3B (henceforth referred to as mDNMT3B) using 2QRV as the template [23].

The estimated absolute model quality for mDNMT3B in terms of *Q*-mean *Z*-score was −2.24, and this modelled structure was subsequently used for docking studies [24]. The X-ray crystal structure of HDAC1 in complex with a fragment of metastasis-associated protein was recently solved at 3 Å resolution and is available at RCSB Protein Data Bank (PDB ID: 4BKX) [25]. The HDAC1 chain (chain B) was extracted and used for the subsequent docking studies.

### 2.7. Defining Substrate Binding Pocket of mDNMT3B and HDAC1

In order to define the substrate binding site in mDNMT3B, the protein was submitted to the CASTp server [26]. The pocket containing both the active residue (Cys-651) and cofactor binding site (Glu-605) was accepted as the substrate binding pocket of mDNMT3B. The pocket was further evaluated by docking of the inhibitor (5-Aza-dC) on the protein structure.  Table 1 lists all the residues that constitute the substrate binding pocket of mDNMT3B. Several of these residues match the active site residues as described by another group [27].

The active site of HDAC1 was defined by comparison with histone deacetylase 8 (HDAC8) where the crystal structure is solved with the inhibitor trichostatin A (TSA) at 1.9 Å resolution (PDB ID: 1T64). The crystal structure contained two molecules of TSA and we have considered the TSA bound in the active site tunnel for our analysis. The interacting residues of HDAC8 with ligand TSA were obtained from LIGPLOT, which uses HBPLUS program to schematically depict the interactions in terms of hydrogen bonds and nonbonded contacts [28]. To identify the corresponding residues of HDAC1, structural alignment of HDAC1 and HDAC8 was performed using the protein structure comparison service PDBeFold at the European Bioinformatics Institute [29]. Root mean square deviation (RMSD) calculated between C*α*-atoms of matched residues at the best 3D superposition of the structures provided a value of 1.1 Å. *Q*-score (range 0-1), which is a measure of the quality of the alignment and takes into account both the RMSD value and the alignment length, was observed to be 0.813 while the *Z*-score was 18.58, both indicative of a high structural similarity.  Table 2 shows the residues of HDAC8 that interacts with TSA in the crystal structure and the equivalent residues of HDAC1 obtained after structural alignment with HDAC8. Binding of SFN in the same cavity where inhibitor TSA binds will be an indication that SFN produces its inhibitory effects by a similar mechanism. The HDAC1 structure was further submitted to CASTp server to identify its complete substrate binding pocket [26]. The largest pocket identified by CASTp included several of the predicted substrate binding residues listed in Table 1 and was therefore defined as the substrate binding pocket. The active site of class I HDAC family has been equated to a tunnel bearing a catalytically pivotal zinc ion towards its end [30]. A previous study on HDAC1 and biarylalanine-containing hydroxamic acids has also referred to a similar set of residues [31].

### 2.8. Docking

Three-dimensional structures of SFN, 5-Aza-dC, and TSA in mol2 format were retrieved from the ZINC database and their structures are depicted in Figure 3 [32]. Blind docking of ligands (SFN) with protein HDAC1 and ligands (SFN and 5-Aza-dC) with protein mDNMT3B was performed using SwissDock, which uses EADock algorithm [33] to identify the binding sites of ligands on the respective proteins. All the residues of the proteins were held fixed and a binding pocket was not defined so as not to bias the docking towards the active site. The parameters selected for docking on the SwissDock server were “accurate” with no flexibility of side chain of any amino acid of the target proteins. Each docking experiment results in many binding modes of the ligand, which are clustered according to their FullFitness scores after calculating their energies using CHARMM. A more favorable binding mode is indicated by a more negative FullFitness score. Analyses of all docked poses were performed using the molecular visualization software UCSF-Chimera [34].

### 2.9. Bisulfite Modification and Methylation-Specific PCR (MS-PCR)

DNA was extracted from HeLa cells after the treatment with SFN at 0, 24, 48, and 72 h, respectively, by using GenElute Mammalian Genomic DNA Miniprep kit (Sigma, USA) as per the manufacturer's instructions. Furthermore, bisulphite modification and purification of DNA samples were carried out by the Imprint DNA Modification kit (Sigma, USA) protocol. The modified DNA was used as a template for MSP (methylation-specific PCR), to distinguish between methylated and unmethylated promoter regions of RAR*β*, CDH1, DAPK1, and GSTP1 genes by using specific primers sets as discussed previously (methylated and unmethylated, resp.) [4, 35–37]. MSP was performed on 50 ng of bisulfite-treated DNA under the following conditions: initial denaturation at 95°C for 5 min, followed by 35 amplification cycles (denaturation at 94°C for 30 s, annealing Tm (RAR*β*: 56°C, CDH1: 56°C, DAPK1: 57°C, and GSTP1: 56°C) for 30 s, and extension at 72°C for 45 s) with final extension at 72°C for 7 min.

### 2.10. Reverse Transcription-PCR

Total RNA isolation was carried out as per the manufacturer's protocol using GenElute Mammalian Genomic Total RNA kit (Sigma, USA) from SFN treated and untreated HeLa cells at various time points (24, 48, and 72 h). Reverse transcription of RNA to synthesize cDNA was performed using the ProtoScriptM-MuLVTaq RT-PCR Kit (New England Bio Labs, USA) from 5 mg of total RNA (at 42°C for 60 min) followed by RT-PCR using gene-specific primers for *β*-actin, RAR*β*, CDH1, DAPK1, GSTP1, DNMTB, and HDAC1. The PCR cycle was as follows: initial denaturation at 95°C for 5 min, followed by 35 amplification cycles (denaturation at 94°C for 30 s, annealing Tm (*β*-actin: 56°C, RAR*β*: 56°C, CDH1: 55.5°C, DAPK1: 56°C, GSTP1: 55°C, DNMT3B: 56°C, and HDAC1: 56°C) for 30 s, and extension at 72°C for 45 s) with final extension at 72°C for 7 min. The primer sequences used were described previously [4, 18, 38–42]. Amplified products were visualized on a 2% agarose gel containing ethidium bromide.

### 2.11. Statistical Analysis

All data are expressed as means ± SD of at least 3 experiments. Fisher's exact test was adopted for statistical evaluation of the results. Significant differences were established at *P* < 0.05.

## 3. Results

### 3.1. SFN Inhibits DNMTs Activity and Downregulates the Expression of DNMT3B in HeLa Cells

**Figure 1:**
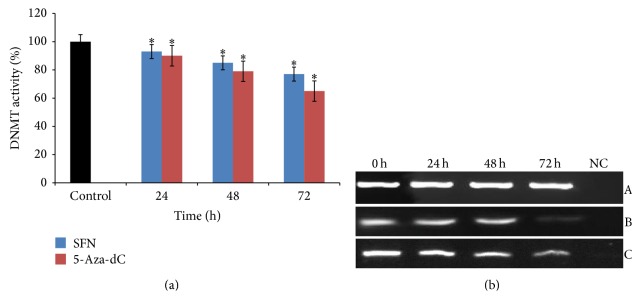
Effect of SFN and 5-Aza-dC on DNMT3B in human cervical cancer cells (HeLa). (a) 2.5 *μ*M of SFN and 1.5 *μ*M 5-Aza-dC treatments significantly inhibit the activity of DNMT in time-dependent manner, respectively. Values are means ± SD of three independent experiments. Symbol (*∗*) indicates significant (*P* < 0.05) difference of data between control and treated cells. (b) 2.5 *μ*M SFN treated HeLa cells show significantly time-dependent reduction in the mRNA expression of DNMT3B in comparison to untreated cells. Panel A shows *β*-actin expression as an internal control, Panel B shows the expression of DNMT3B on treatment with 5-Aza-dC, and Panel C shows the expression of DNMT3B on treatment with SFN. Lane 1 shows the expression of DNMT3B gene in untreated HeLa cells; Lanes 2, 3, and 4 show the time-dependent alteration in the expression of DNMT3B after treatment for 24, 48, and 72 h, respectively; Lane 5 shows negative control for RT-PCR.

DNA methyltransferase (DNMT) activity assay was performed in nuclear extract, extracted from SFN treated HeLa cells at various time points (24, 48, and 72 h). SFN was found to exert significant time-dependent inhibition of DNMT activity (7%, 15%, and 23%) in HeLa cells compared with the untreated control (Figure 1(a)). Time-dependent (24, 48, and 72 h) exposure of HeLa cells with 1.5 *μ*M 5-Aza-dC resulted in 10%, 21%, and 35% inhibition of DNMT activity in comparison to untreated control. Furthermore, whether the activity of DNMT correlated with the expression of DNMT3B induced by SFN treatment in HeLa cells was determined. Untreated HeLa cells exhibited the highest levels of DNMT3B mRNA whereas SFN treated cells showed significant decrease in the expression of DNMT3B in a time dependent-manner (24, 48, and 72 h) (Figure 1(b)). Similarly, 5-Aza-dC treated cell also showed time-dependent decrease in the expression of DNMT3 B. *β*-actin was used as an internal control for comparison of samples (Figure 1(b)).

### 3.2. SFN Inhibits HDACs Activity and Reduces the Expression of HDAC1 in HeLa Cells

**Figure 2:**
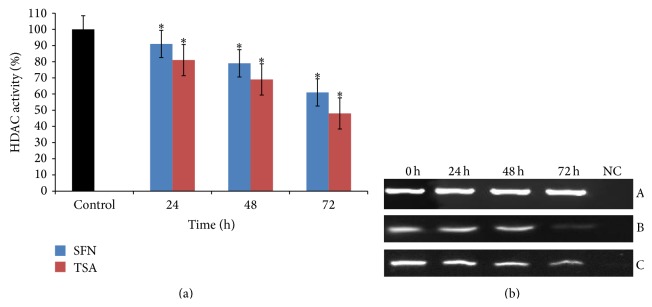
Effect of SFN and TSA on HDAC1 in human cervical cancer cells (HeLa). (a) 2.5 *μ*M of SFN and 0.05 *μ*M TSA treatments significantly inhibit the activity of HDAC in time-dependent manner, respectively. Values are means ± SD of three independent experiments. Symbol (*∗*) indicates significant (*P* < 0.05) difference of data between control and treated cells. (b) 2.5 *μ*M SFN treated HeLa cells show significantly time-dependent reduction in the mRNA expression of HDAC1 in comparison to untreated cells. Panel A shows *β*-actin expression as an internal control, Panel B shows the expression of HDAC1 on treatment with TSA, and Panel C shows the expression of HDAC1 on treatment with SFN. Lane 1 shows the expression of HDAC1 gene in untreated HeLa cells; Lanes 2, 3, and 4 show the time-dependent decrease in the expression of HDAC1 after treatment for 24, 48, and 72 h, respectively; Lane 5 shows negative control for RT-PCR.

**Figure 3:**
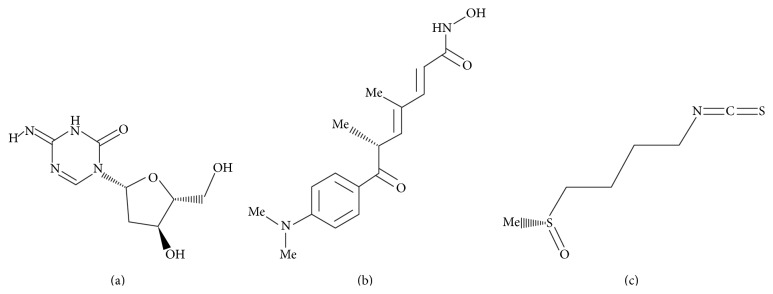
Structures of the ligands used in the docking study. (a) 5-Aza-dC. (b) TSA. (c) SFN.

HDACs are an enzyme family which is mainly involved in histone deacetylation and linked with the increased levels of epigenetically silenced genes in cancer. The activity of HDACs in HeLa cells was determined by treating the cells with SFN at 24, 48, and 72 h, respectively. It was observed that SFN treated HeLa cells showed a time-dependent decline of 9%, 21%, and 39% in HDACs activity (Figure 2(a)). It was also observed that the exposure of HeLa cells to HDAC inhibitor (0.05 *μ*M TSA) showed time-dependent decrease in the activity of HDACs and caused 24% inhibition after 24 h of exposure (Figure 2(a)). Moreover, whether the decline in HDACs activity correlated with a decrease in HDAC1 expression was also investigated. It was found that exposure of HeLa cells with SFN showed a significant time-dependent decrease in the expression of HDAC1 in comparison to untreated cells (Figure 2(b)). Similar results were noticed after treatment of HeLa cells with TSA and showed significant reduced expression of HDAC1 at 24, 48, and 72 h, respectively (Figure 2(b)).

### 3.3. SFN Interacts with DNMT3B and HDAC1: An* In Silico* Theoretical Molecular Modeling

*In silico* theoretical molecular modeling approach was used to investigate the possible mechanism by which SFN inhibits DNMT3B and HDAC1. Substrate binding site of DNMT3B was defined as that predicted cavity which included the active site residue Cys-651 and cofactor binding residue Glu-605. The cavity was further evaluated by docking of a well-known DNMT3B inhibitor 5-Aza-dC on mDNMT3B using SwissDock server. Substrate binding site of HDAC1 was defined as that cavity predicted by CASTp which included the HDAC1 residues equivalent to those of HDAC8 interacting with its ligand TSA in the crystal structure. This cavity also included the active site His-141 and Zn ion. Using the same docking server, SFN was docked on HDAC1 and mDNMT3B. In the docking experiments by SwissDock, FullFitness and Gibbs free energy (Δ*G*) of each run (256 runs) of the docking were evaluated. Favorable binding modes were scored based on FullFitness and cluster formation. The value of FullFitness was used to rank clusters for further analysis.

### 3.4. SFN Interaction with mDNMT3B

The docking of 5-Aza-dC, a well-known inhibitor of DNMT3B, was performed first to define the substrate binding site of the protein. The docking results produced 43 clusters of ligands around the modelled catalytic domain of DNMT3B. Fifteen out of these 43 clusters bind in the CASTp predicted cavity which includes the active site Cys-651 and cofactor (S-adenosyl methionine) binding residue, Glu-605. The ligand models in the top 2 clusters (0 and 1) containing 15 elements were indeed very close to these active site residues (Table 3). The distances of the closest atoms of Cys-651 and Glu-605 to the most favorable docked model of 5-Aza-dC are 1.86 and 2.34 Å, respectively. Thus the substrate binding cavity of mDNMT3B predicted by CASTp was found to be in agreement with the docking result of 5-Aza-dC on the protein. Next, the docking of SFN was performed on mDNMT3B to probe if SFN also binds to the same substrate binding cavity.

**Figure 4:**
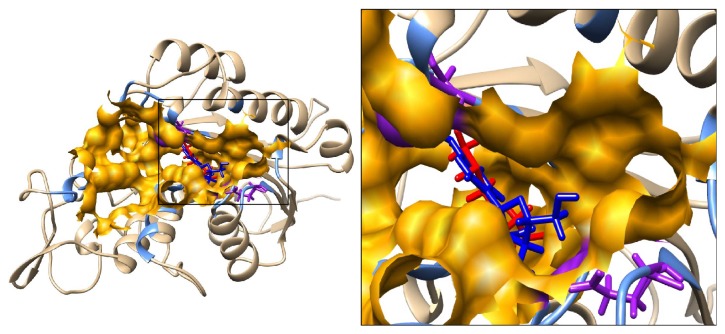
Predicted interaction between ligands (SFN and 5-Aza-dC) with mDNMT3B. The mDNMT3B is depicted in ribbon representation showing docked models of SFN in red and 5-Aza-dC in blue and the residues defining the pocket as light blue. Inset focuses on the binding pocket shown in orange. Active site C-651 and cofactor binding E-605 are labeled and shown in purple solid bonds.

The docking results produced 49 clusters of the ligand SFN around the modelled catalytic domain of DNMT3B. Analysis of these clusters showed that 31 of these 49 clusters bind in the substrate binding cavity as defined by CASTp. These clusters together contained a total of 188 elements out of 256 predicted binding modes. Interestingly, the top 9 clusters (from 0 to 8) containing a total of 86 elements were in this cavity. Table 3 shows the summary result of SwissDock docking with the FullFitness and estimated Δ*G* values for the most favorable interaction. Observation of the majority of the clusters, including the top ranked ones in the cavity, strongly suggests that the preferred binding of SFN on mDNMT3B is within the substrate binding cavity and overlaps with binding site of 5-Aza-dC.  Figure 4 shows the visualization of the most energetically favorable binding of SFN and 5-Aza-dC on the protein mDNMT3B.  Table 4 lists all the mDNMT3B residues within 5 Å of the most energetically favorable docked model of SFN.

### 3.5. SFN Interaction with HDAC1

**Figure 5:**
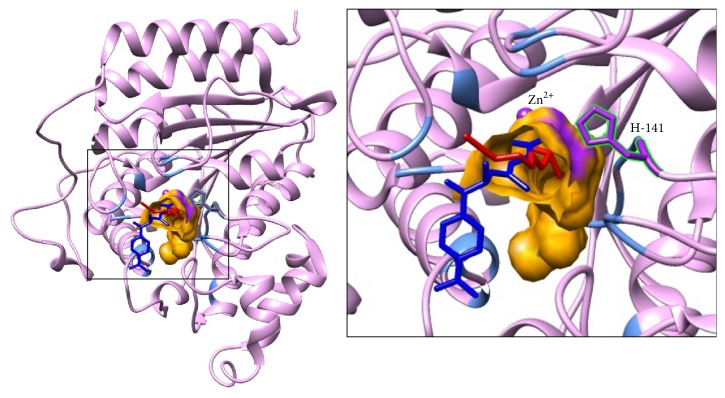
Predicted interaction between ligands (SFN and TSA) with HDAC1. The HDAC1 protein is depicted in ribbon representation showing docked model of SFN in red and TSA in blue and the residues defining the pocket as light blue. The TSA structure was transformed from HDAC8 by superimposition on HDAC1. Inset focuses on the binding pocket shown in orange. The active site H-141 and Zn ion are labeled and highlighted in purple.

The docking results produced 41 clusters of ligand SFN around the complete protein HDAC1. Analysis of these clusters showed that 6 of these clusters bind in the substrate binding cavity. These clusters together contained a total of 40 elements out of 256 predicted binding modes. Interestingly, these clusters included the top ranked clusters 0, 1, 2, and 3 in addition to other clusters with ranks 16 and 19. Table 3 shows the SwissDock docking result and the FullFitness and estimated Δ*G* values for the most favorable interaction. The lowest energy model of cluster rank zero is considered to be the most favorable interaction. Observation of top clusters (0, 1, 2, and 3) in the cavity strongly suggests that the preferred binding of SFN on HDAC1 is within the substrate binding cavity.  Figure 5 shows the visualization of the most energetically favorable binding of SFN on the protein HDAC1. The figure also shows the binding of TSA on the active site of HDAC1 which was obtained after superimposition of the crystal structure of HDAC8 bound to TSA on the structure of HDAC1. It can be clearly seen that predicted binding of the most favorable SFN and transposed TSA overlaps the same binding region on HDAC1. The active site His-141 and Zn ion which is known to play crucial catalytic roles are lining the cavity and within 5 Å of the ligands.  Table 4 lists all the HDAC1 residues within 5 Å of the most energetically favorable docked model of SFN.

### 3.6. SFN Mediated Methylation Reversal Reactivates or Increases the Expression of RAR*β*, CDH1, DAPK1, and GSTP1 Genes in HeLa Cells

**Figure 6:**
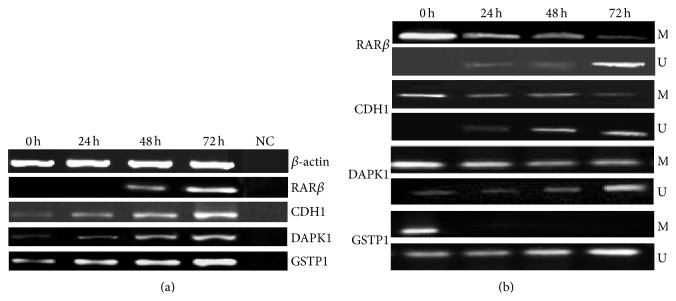
Alterations of methylation status and mRNA expression levels of RAR*β*, CDH1, DAPK1, and GSTP1 genes after treatment with SFN. (a) mRNA expression levels before and after the treatment. Lane 1 shows the expression of these genes in untreated HeLa cells; Lanes 2, 3, and 4 show the time-dependent modulation in the expression of HDAC1 upon treatment for 24, 48, and 72 h, respectively; Lane 5 shows negative control for RT-PCR. *β*-actin was used as an internal control. (b) Methylation-specific bands (M) and unmethylation-specific bands (U). Lane 1 shows the methylation status of these genes in untreated HeLa cells; Lanes 2, 3, and 4 show the time-dependent modulation in the methylation status of RAR*β*, CDH1, DAPK1, and GSTP1 genes for 24, 48, and 72 h, respectively.

To establish whether SFN induced inhibition of DNMT3B and HDAC1 leads to reexpressing or increasing the expression of RAR*β* (retinoic acid receptor beta), CDH1 (cadherin 1), DAPK1 (death associated protein kinase 1), and GSTP1 (glutathione S-transferase P1) genes, expression studies of these genes were performed. Expression of these genes was further correlated with the changes in the methylation of their promoter regions. It was observed that time-dependent exposure (24, 48, and 72 h) of HeLa cells with SFN resulted in a significant increase in the expression of RAR*β*, CDH1, DAPK1, and GSTP1 genes in comparison to untreated cells (Figure 6(a)). Interestingly, it was observed that the expression of these genes is associated with the 5′CpG dinucleotide island methylation or unmethylation of their promoter regions. In this study, we analyzed the 5′CpG island methylation of RAR*β*, CDH1, DAPK1, and GSTP1 genes by using MS-PCR in SFN treated HeLa cells and compared them with untreated cells.

Treated cells were subjected to MSP with methylation-specific and unmethylation-specific sets of primers. Treated cells showing amplification only after using methylated and unmethylated primers set were considered as hypermethylated and unmethylated, respectively, while treated cells showing amplification using both sets of primers were marked as hypomethylated. From the MSP results, it was observed that RAR*β*, CDH1, and DAPK1 genes were found to be hypermethylated while GSTP1 gene was found to be hypomethylated in untreated HeLa cells as RAR*β*, CDH1, and DAPK1 genes promoters were amplified only with the methylation-specific primers set and GSTP1 was amplified with both methylated and unmethylated primers set. However, upon treatment with SFN, the methylated state was reversed, as this was evident from the decreased level of amplimer intensity with methylation-specific primers, whereas it was significantly increased with unmethylated set of primers in a time-dependent manner (Figure 6(b)). Hence, this confirms that treatment with SFN reverses the expression of RAR*β*, CDH1, and DAPK1 genes but increases the expression of GSTP1 through the reversal in the 5′-CpG island methylation and by inhibiting the activity of epigenetic modulators like DNMT3B and HDAC1 in HeLa cells.

## 4. Discussion

Epigenetic modulations such as DNA methylation and histone modifications are considered principal epigenetic events and have been acknowledged for the silencing of many genes including tumor suppressor genes (TSGs) that lead to cancer development and progression. The dynamic and reversible nature of the epigenetic processes makes them a potential target to of reverse the process carcinogenesis [8, 10, 15, 43–45]. Epigenetic regulation of TSGs expression is meditated by key enzymes such as DNA methyltransferases (DNMTs) and histone deacetylases (HDACs). Use of classical DNMTs and HDACs inhibitors induces reversal of epigenetic modifications via DNA demethylation and histone acetylation, respectively, leading to reactivation of silenced genes; however, clinical efficacy of these inhibitors has been somewhat limited due to the side effects. Consequently, in searching of potential DNMTs and HDACs inhibitors, studies on dietary phytochemicals are gaining interest to develop safe epigenetic drugs. In the present study, we studied epigenetic regulation by SFN, a potent isothiocyanate, and its anticarcinogenic, anti-inflammatory, and antioxidative effects have been established in earlier and other studies [18, 46–48].

Previous studies in our lab indicated that SFN was found to be selectively cytotoxic towards cancer cells and inhibit the growth of cancer cells in a dose- and time-dependent manner and EC_50_ of SFN on HeLa cells was found to be 12 *μ*M [18]. From these results, we selected a sublethal dose of SFN (2.5 *μ*M) for the present study. Due to the integral cytotoxicity of classical DNMTs and HDACs inhibitors, it is important to identify novel therapeutic agents which can modulate the epigenetic process through blocking the DNMTs and HDACs without destabilizing the genome and possessing a safe therapeutic profile. Pertinent to this exploration, it was observed that SFN treated HeLa cells resulted in the inhibition of the activity of DNMT and HDAC enzymes in a time-dependent manner (Figures 1(a) and 2(a)). In addition, treatment of HeLa cells with 5-Aza-dC (1.5 *μ*M), a well-known DNA methylation inhibitor, and TSA (0.05 *μ*M), a well-recognized HDAC inhibitor, was found to have almost similar inhibitory effect on the enzyme activity of DNMT and HDAC, respectively, in a time-dependent manner (Figures 1(a) and 2(a)). Individual effect of 5-Aza-dC and TSA at various doses on DNMTs and HDACs activity on HeLa cells is not shown (unpublished). Interestingly, it was observed that SFN treated HeLa cells showed significant time-dependent decrease in the expression of mRNA transcripts of DNMT3B and HDAC1 in comparison to untreated cells (Figures 1(b) and 2(b)). Our study provides the first evidence that SFN induces epigenetic modulation through the inhibition of DNMTs activity and downregulation of DNMT3B in HeLa cells whereas many studies have shown that 5-Aza-dC inhibits DNMT enzymatic activity rather than expression of DNMTs [49–51]. Our results are consistent with other studies in which SFN treatment downregulates the expression of DNMT3B and HDAC1 [52, 53].* In vivo* and* in vitro* studies have shown that SFN treatment for breast cancer, normal, hyperplastic, and cancerous prostate cells, human mesenchymal stem cells, nasopharyngeal carcinoma cells, colon cancer cells, and porcine satellite cells was found to significantly inhibit the activity of HDACs [9, 11, 46–48, 52–56]. Similarly, TSA treatment of many cancer cells resulted in the loss of HDACs enzymatic activity [57, 58].

To know whether SFN mediated enzymatic inhibition of DNMTs and HDACs is due to direct binding of it with these enzymes,* in silico* molecular modeling studies were performed to identify the interaction of SFN with modelled DNMT3B and HDAC1. Our analyses of the predicted docking results indicate that SFN directly binds in the substrate binding pocket of the enzymes. Computational and knowledge-based approaches were used to define the substrate binding pocket of DNMT3B. Docking of 5-Aza-dC in conjunction with knowledge about active sites and cofactor binding sites helped us to specify the substrate binding pocket for DNMT3B.  Table 4 lists these pocket lining residues which included active sites E-605 and C-651 important for binding to SAM (S-adenosyl methionine), which is critical for methyl transferase activity.  Figure 4 illustrates the best energetically favored models of SFN and 5-Aza-dC docked on mDNMT3B. Interestingly, a very high proportion (31 out of 49, containing 188 out of 256 independent docking runs) of predicted top ranked clusters for SFN was observed in the defined substrate binding pocket of mDNMT3B.

These results show that SFN and 5-Aza-dC overlap the same site in the protein and therefore may have a similar mechanism of protein inhibition by preventing the entry of the natural ligand into the active site. The proximity of SFN and 5-Aza-dC to the active residue Cys-651 and the cofactor binding residue Glu-605 adds emphasis to this hypothesis. This leads us to propose that, given the better safety profile of SFN in comparison to 5-Aza-dC, SFN is a better candidate as a similarly functioning epigenetic modulator. Other groups have reported on the inhibition of DNMT3B by nanomycin A following a similar pattern and involving the residues Asp-697, Arg-731, and Arg-733 [27]. These residues are also included in the pocket defining residues for our studies. In Table 4 we list those residues of DNMT3B which interact with the most energetically favorable docked model of SFN.

As shown in Figure 5, the best energetically favored model of SFN docked on HDAC1 overlaps with the binding site of TSA which was observed in the crystal structure of HDAC8. As summarized in Table 3, several top scoring docking clusters, containing many elements of independent docking, predict binding of SFN in the same region. The results suggest that HDAC1 activity could be inhibited directly by SFN with the observed interactions which could block the entry of the cognate substrate and its subsequent catalysis. High sequence and structural similarity between HDAC8 and HDAC1 strongly suggest that the substrate binding region is very similar between the two HDACs. As shown in Table 2, based on the conserved sequences identified in HDAC8 which are involved in the catalytic activity, we expect that equivalent residues of HDAC1 are also important for its catalytic activity. Of note, His-141 and Zn^2+^ are located at the end of the active site tunnel and have been shown to play a crucial role in the catalytic activity of HDAC8 by having interactions with its natural substrate or inhibitor molecules such as SAHA during the catalytic action [59]. An earlier study also indicates that Asp-176 and Asp-183 may be important residues for the catalytic action of HDAC8 [59]. The structurally equivalent residues important for HDAC1 activity would be Asp-174 and Asp-181 [59]. Furthermore, we list other residues of HDAC1 interacting with the most energetically favorable docked model of SFN in Table 4. Our molecular modeling and docking studies not only successfully explain the mechanism of action of SFN in inhibiting the epigenetic modulating enzymes but also pave the way to explore further avenues such as structure-guided optimization studies and pharmacophore modeling.

The possible epigenetic effects of SFN on HeLa cells to increase the expression or reactivation of epigenetically silenced tumor suppressor genes (TSGs) including RAR*β*, CDH1, DAPK1, and GSTP1 expression were also correlated with its ability to inhibit DNMTB and HDAC1 activity. Probably, inhibition of DNMTs and HDACs favors a decrease in the hypermethylation and silencing of these key genes and, therefore, this inhibitory action of SFN may contribute to cancer prevention. The aberrant promoter methylation leads to reduced expression in various TSGs and results in tumorigenesis [60–62]. Extensive studies have reported that RAR*β*, CDH1, DAPK1, and GSTP1 genes are transcriptionally silenced not only through mutation and genomic instabilities (LOH, microsatellite instabilities, and homozygous deletions) but also by lack of expression due to promoter hypermethylation during the development of various human cancers [60–71]. In the present study, it was observed that RAR*β*, CDH1, and DAPK1 genes were found to be hypermethylated and correlated with their respective expression which was found to be undetectable whereas GSTP1 gene was found to be hypomethylated and its expression was significantly detectable in untreated HeLa cells (Figures 6(a) and 6(b)). However, after SFN treatment, HeLa cells showed time-dependent changes in the methylation status of RAR*β* and DAPK1 genes as indicated both by the reduction in the bands of the methylation panel, and with significantly detectable bands in the unmethylated panel. This change can be linked with the restoration of the expression of RAR*β* and DAPK1 genes over the time exposure to SFN. Whereas, hypomethylated GSTP1 genes were found to be unmethylated and their expression increased in a time-dependent manner (Figures 6(a) and 6(b)). The result of our study is similar to several other studies which have shown that a variety of dietary agents including SFN reactivate many TSGs via modulation of various epigenetic pathways [40, 44, 52, 72, 73].

In summary, it can be inferred that SFN may act as potential chemopreventive agent and modulates epigenetic events via inhibition of the activity of DNMTB and HDAC1 and may reactivate epigenetically silenced TSGs by altering methylation status of these genes promoter regions. SFN can be used as effective inhibitors of DNMTs and HDACs to prevent cancer. Furthermore, use of SFN, as epigenetic modifier, in animal models or humans remains to be demonstrated.

## Figures and Tables

**Table 1:** Residues defining the substrate binding pocket of HDAC1 and mDNMT3B. Active site residues are underlined.

Protein	Residues lining the substrate binding cavity
mDNMT3B	C-651, E-605, F-581, D-582, G-583, T-586, S-604, E-605, V-606, C-607, V-628 G-648, S-649, P-650, C-651, N-652, S-655, V-657, N-658, P-659, L-671, E-697 V-699, V-700, A-701, R-731, A-732, R-733, R-773, I-774, K-777, S-778, N-779, S-780 I-781, R-823, G-824, Q-827, K-828, G-831, R-832, S-833, W-834

HDAC1	H-140, H-141, D-176, H-178, D-264, L-271, F-109, W-135, A-136, G-137, L-139, G-149, C-151, F-205, Zn ion

**Table 2:** Comparison of substrate binding residues of HDAC8 and HDAC1.

HDAC8	Y	D	H	H	G	F	D	D	H	D	D	M	Y
	100	101	142	143	151	152	176	178	180	183	267	274	306

HDAC1	E	D	H	H	G	F	D	D	H	D	D	L	Y
	98	99	140	141	149	150	174	176	178	181	264	271	303

**Table 3:** Docking results of ligands (SFN, TSA, and 5-Aza-dC) on receptors (HDAC1 and mDNMT3B).

Receptor	Ligand	Clusters within substrate binding cavity/total clusters	Cluster ranks	Total elements (out of 256)	FullFitness (kcal/mol)	Estimated Δ*G* (kcal/mol)
HDAC1	SFN	6/41	0, 1, 2, 3, 16, 19	40	−2094.8	−7.9

mDNMT3B	SFN	31/49	0–8, 10, 12–16, 18, 21–27, 29–31, 33–34, 43, 45	174	−1914.2	−7.5

mDNMT3B	5-Aza-dC	15/43	0–1, 3–5, 7, 17, 19, 24, 34, 36–37, 39, 42, 43	73	−2071.4	−9.5

**Table 4:** Residues of HDAC1 and mDNMT3B within 5 Å of SFN.

Protein	Residues within 5 Å of SFN
HDAC1	M-30, L-139, H140, H141, F150, C-151, D-176, H-178, F-205, D-264, L271, G-300, G-301, Y-303, Zn ion

mDNMT3B	F-581, D-582, G-583, T-586, G-648, S-649, P-650, C-651, E-697, V-699, R-733, R-832, S-833, W-834
